# Effects of Postoperative Radiotherapy in Early Breast Cancer Patients Older than 75 Years: A Propensity-Matched Analysis

**DOI:** 10.7150/jca.35204

**Published:** 2019-10-17

**Authors:** Linghui Zhou, Pengtao Yang, Yi Zheng, Tian Tian, Cong Dai, Meng Wang, Shuai Lin, Yujiao Deng, Qian Hao, Zhen Zhai, Hongtao Li, Zhijun Dai

**Affiliations:** 1 Department of Breast Surgery, The First Affiliated Hospital, School of Medicine, Zhejiang University, Hangzhou 310003, Zhejiang, China.; 2 Department of Oncology, The 2nd Affiliated Hospital of Xi'an Jiaotong University, Xi'an 710004, Shaanxi, China.; 3 Department of Breast Head and Neck surgery, The 3rd Affiliated Teaching Hospital of Xinjiang Medical University (Affiliated Tumor Hospital), Urumqi 830000, Xinjiang, China.

**Keywords:** postoperative radiotherapy, early breast cancer, seer database, propensity score matching, prognosis

## Abstract

**Background**: Currently, there is still some controversy regarding whether early breast cancer patients with a tumor size of ≤5 cm and 1-3 positive lymph nodes should undergo postoperative radiotherapy (PRT).

**Materials and Methods**: We obtained data from the Surveillance, Epidemiology, and End Results (SEER) 18 database. Then, we conducted propensity score matching (PSM), according to the radiotherapy record. The Kaplan-Meier and Cox regression analysis were conducted to explore prognostic factors in breast cancer.

**Results**: A total of 6,777 patients aged 75+ years old were eligible and 2,361 patients were included after PSM. We found PRT could improve patient overall survival (OS) (*P* = 0.01, hazard ratio [HR] = 0.88, 95% confidence interval [CI], 0.80-0.97). Subgroup analysis revealed PRT could improve OS in patients with hormone receptor positive (HR+) (*P* = 0.001, HR = 0.84, 95% CI, 0.76 - 0.94) or white patients (*P* =0.004, HR = 0.86, 95% CI, 0.77 - 0.95).

**Conclusions**: PRT may benefit for elderly women with early breast cancer, especially in HR+ patients or white patients. These findings may inform future optimized options whether elderly female patients with early breast cancer should undergo postoperative radiotherapy.

## Introduction

Reportedly, breast cancer is the most commonly diagnosed cancer and accounts for 30% of new cancers diagnosed amongst American females. In 2017, a total of 252,710 people were newly diagnosed with breast cancer and 40,610 females died of breast cancer 1. Due to increased life expectancy and early breast cancer screening programs, there is a rapid increase in the incidence of breast cancer in elderly patients in both developed and developing countries 2. For example, in the United States, elderly patients ≥60 years old account for more than 40% of deaths due to breast cancer 1. It is well known that surgery and radiotherapy are the main treatment strategies for breast cancer in regional lymph nodes. The purpose of surgery is to remove the tumor and regional lymph nodes. However, surgery cannot guarantee complete removal of cancer cells from the skin, chest wall, and lymph nodes, which might increase the risk of relapse. A meta-analysis suggested radiotherapy could reduce the 10-year risk of any first recurrence and the 15-year risk of breast cancer death 3. The role of PRT is still controversial when the tumor size is ≤5 cm and the number of positive lymph nodes is between one and three 4-6. Certain studies suggest that radiotherapy may impair the quality of life in elderly patients 7, 8. A trial of PRT in minimum-risk elderly patients showed that most elderly breast cancer patients can tolerate PRT well without impairing their overall health-related quality of life 9.

Radiotherapy exerts cytotoxic effects by generating free radicals in the target tissue. These free radicals are the source of reactive oxygen species and reactive nitrogen species that produce DNA damage by forming single stranded DNA breaks (SSB) and double stranded DNA breaks (DSB) 10. Due to the specificity of breast cancer cells, differences in chemotherapy and endocrine therapy between different subtypes have been confirmed 11. Similarly, Mao *et al* found that sensitivity to radiotherapy is different among breast cancer subtypes 12. *In vitro* cell experiments also show that breast cancer cells of different subtypes have different sensitivity to ionizing radiation 10.

## Materials and Methods

### Patients

**Figure 1:**
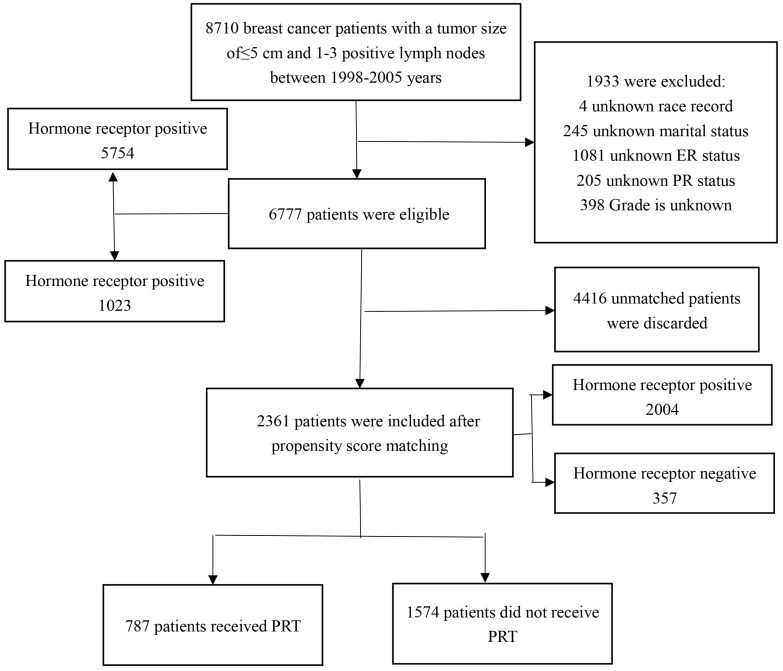
Flow chart of study patients' enrollment. PRT: postoperative radiotherapy.

We obtained patient data from SEER 18 Regs Custom Data (with additional treatment fields), Nov 2017 Sub (1973-2015 varying), using SEER*Stat, version 8.3.5. A total of 6,777 patients were eligible and 2,361 patients were included after PSM. The flow chart for selecting research samples is shown in the **Figure 1**. The following variables were used in the analysis: age, race, marital status, months of survival, vital status records, cause-specific death classification, AJCC T, number of positive regional nodes, ER, PR, grade, laterality, radiation record, and sugery.

### Statistical analyses

In order to improve the evidence level of the test and control for known variables except for PRT on the experimental, 1:2 patient pairing with a caliper size of 0.1 was performed by PSM. Patients aged 75 years who had undergone surgery were divided into two groups according to whether or not PRT was given. We used frequencies and proportions for categorical variables to describe the characteristics of patients and compared the difference of two groups using the chi-square (χ^2^) test. To evaluate the effect of radiotherapy, Kaplan-Meier analysis and log-rank test were conducted. A Cox proportional hazards regression model was conducted to predict independent risk factors for all-cause and breast cancer-specific death. All statistical tests were two-sided, with statistical significance evaluated at the 0.05 αlevel. All calculations were performed by R software (version 3.5.1).

## Results

### Patient demographics

A total of 6,777 patients were identified from SEER 18 Regs Custom Data (with additional treatment fields), Nov 2017 Sub (1973-2015 varying), according to the above-mentioned inclusion criteria. The median survival time for all patients was 89 months (range, 0 - 215 months). A total of 5316 (78.44%) patients died of all causes and 1147 (16.92%) died of breast cancer. Of the included patients, 2481 (36.61%) patients received PRT and 4296 (63.39%) did not receive PRT. There were significant differences in age (*P* <0.001), marital status (*P* <0.001), lymph node (*P* <0.001), AJCC.T (*P* <0.001), ER status (*P* <0.001), PR status (*P* <0.001), grade (*P* <0.001), and surgery (*P* <0.001) between the patients receiving PRT and the patients not receiving PRT. As for race (*P* = 0.505) and laterality (*P* = 0.746), no significant difference was found. The details are listed in **Table 1**.After PSM, all characteristics between the two groups were perfectly balanced. The details are listed in **Supplemental Table S1**.

### Prognostic value of PRT on OS

**Figure 2:**
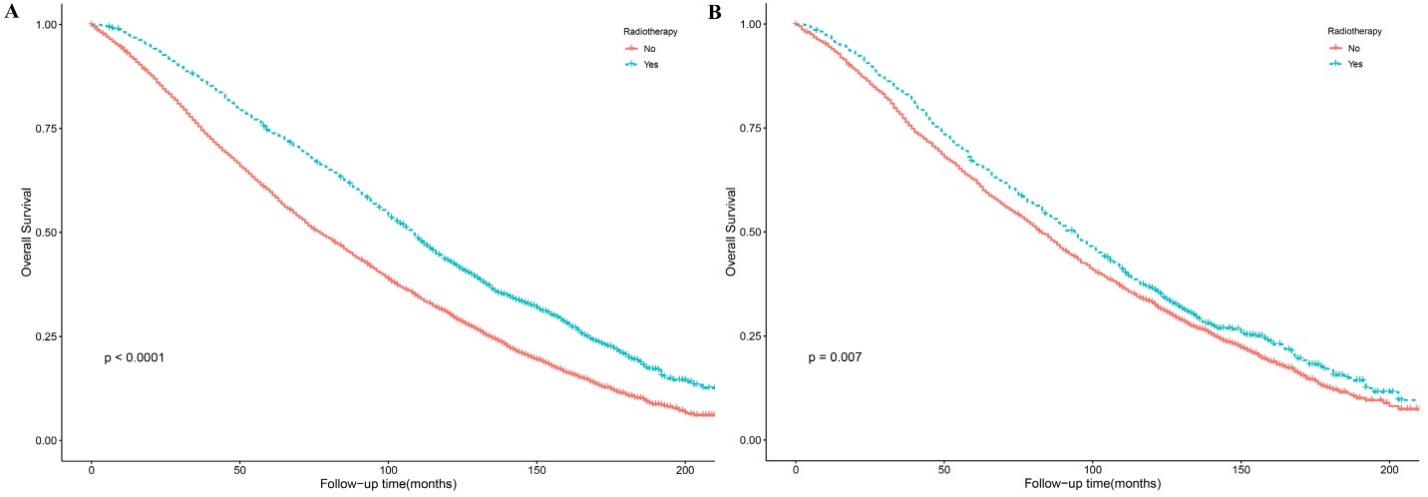
Kaplan-Meier survival curves in early breast cancer patients with age >= 75 Years for overall survival before (A) and after (B) PSM. PSM: propensity score matching.

**Figure 3:**
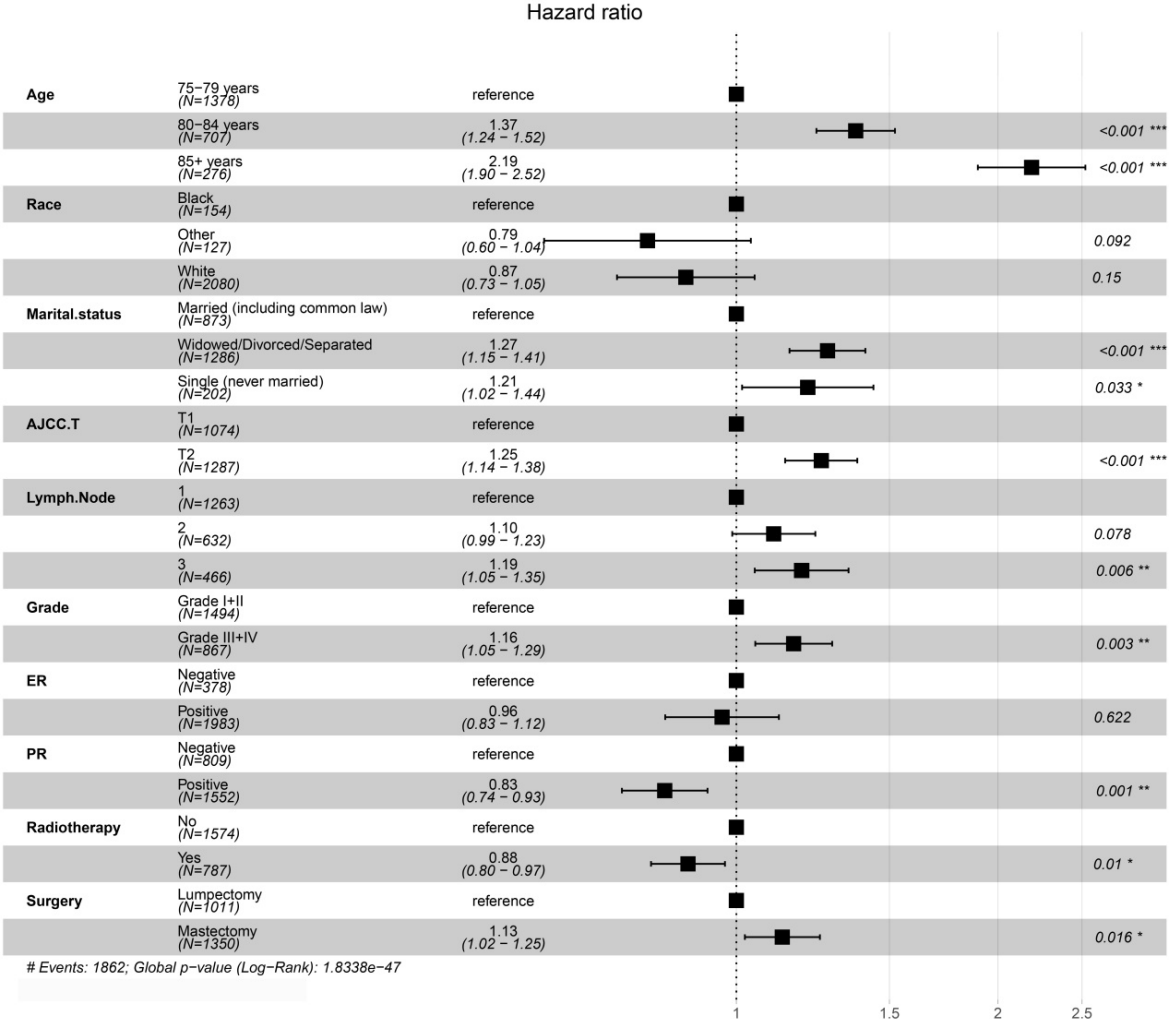
Forest plots of multivariate Cox regression analysis for overall survival in matched patients.

The results of Kaplan-Meier method and log-rank test showed that PRT could improve patient OS before and after PSM (**Figure 2**). We conducted a univariate analysis and found that all the factors (except for laterality) for OS were significant, and then included all the factors into the multivariate Cox regression for analysis. The results of the Cox proportional hazards regression model showed that age, marital status, AJCC T, PR, grade, surgery and radiotherapy are independent risk factors for overall survival. Patients aged 80-84 years-old compared with those aged 75-79 years-old had an HR of 1.37 (*P* <0.001, 95% CI, 1.24 - 1.52); patients aged ≥85 years-old compared with those aged 75-79 years-old had an HR of 2.19 (*P* <0.001, 95% CI, 1.90 - 2.52). Compared with married patients, widowed/divorced/separated patients had an HR of 1.27 (*P* <0.001, 95% CI, 1.15 - 1.41) and single people (never married) had an HR of 1.21 (*P* = 0.033, 95% CI, 1.02 - 1.44). Patients with T2 stage compared with patients with T1 stage had an HR of 1.25 (*P* <0.001, 95% CI, 1.14 - 1.38). Compared with patients with one positive lymph node, patients with three positive lymph nodes had an HR of 1.19 (*P* = 0.006, 95% CI, 1.05 - 1.35). PR+ patients had an HR of 0.83 (P =0.001, 95% CI, 0.74 - 0.93) compared with PR- patients. Patients with grade Ⅲ-Ⅳ compared with grade I-II breast cancer had an HR of 1.15 (*P* < 0.001, 95% CI, 1.07 - 1.24). The patients receiving PRT compared with those not receiving PRT had an HR of 0.88 (*P* =0.01, 95% CI, 0.80 - 0.97). The patients receiving mastectomy treatment compared with those receiving lumpectomy treatment had an HR of 1.13 (*P* =0.016, 95% CI, 1.02 - 1.15). Details were shown in **Table 2 and Figure 3**.

### Subgroup analysis stratified by race and HR status

**Figure 4:**
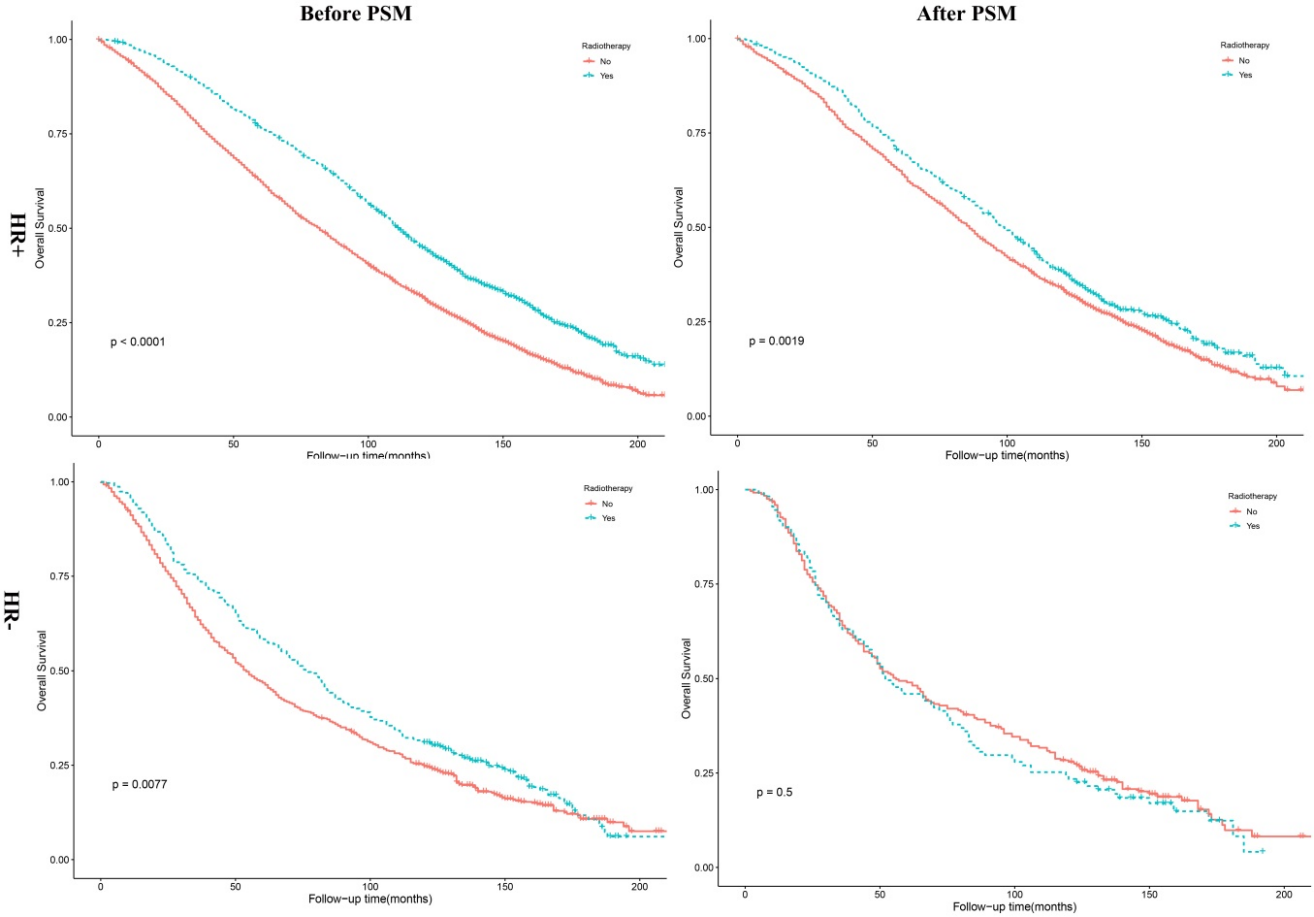
Kaplan-Meier survival curves for overall survival stratified by HR status before and after PSM. PSM: propensity score matching; HR+: hormone receptor positive; HR-: hormone receptor negative.

The results of Kaplan-Meier method and log-rank test revealed that HR+ patients underwent PRT had a better OS (**Figure 4**, *P* =0.0019). As shown in **Table 3**, this was in accordance with the results of multivariate Cox analysis before (*P* <0.001, HR = 0.80, 95% CI, 0.74 - 0.87) and after PSM (*P* =0.001, HR = 0.84, 95% CI, 0.76 - 0.94). However, the results of multivariate Cox analysis revealed no association between PRT and HR- patients (*P* = 0.62, HR = 1.05, 95% CI, 0.87 - 1.27). In addition, we found patients receiving PRT had a better and OS (*P* =0.002) among white patients, compared with patients not receiving PRT. The results of multivariate analysis also showed that patients receiving PRT had a better OS among white patients (*P* = 0.004, HR = 0.86, 95% CI, 0.77 - 0.95). Details were shown in **Table 4.**

### Sensitivity analysis

To verify the stability of our results, we also conducted a multivariate Cox regression analysis of unmatched patients for OS. The results of multivariate Cox regression analysis of unmatched patients were basically consistent with those of matched patients. The details were listed in **Supplemental Table S2**.

## Discussion

To our knowledge, this study is the first research to explore the role of elderly female breast cancer patients older than 75 years old with a tumor size of ≤5 cm and 1-3 positive lymph nodes, based on SEER database. We found that PRT may benefit for elderly women with early breast cancer, especially in HR+ patients or white patients.

Age was an important indicator for overall survival, and there was an imbalance in the age distribution of patients who received PRT (9.47%, ≥85-years-old) and those who did not receive PRT (20.33%, ≥85-years-old). The number of patients receiving radiation therapy decreased with age, which might be due to the lowering of expectations of survival for the elderly. However, there was no clear evidence that PRT was not beneficial to this population. We found an inverse association between the receipt of postoperative radiotherapy and age. Moreover, patients who received postoperative radiotherapy, on average, tended to have a higher OS. This is consistent with the research conducted by Ali et al 13. As the average life expectancy in the United States continues to increase, the standard for “elderly” care may continue to rise. We chose 75 years old as the distinction for elderly patients compared to previous research using 70 years old and was consistent with the latest National Comprehensive Cancer Network (NCCN) clinical practice guidelines 14.

In the 13th St Gallen International Breast Cancer Conference, 64% of experts believed that PRT should not be used routinely in breast cancer patients with T1-2N1M0, but two-thirds of experts felt that PRT should be given to patients with poor prognosis 5. A randomized trial study by Holli et al found that PRT does not increase OS in breast cancer patients 15. Similarly, Tang et al also suggested PRT should be considered with caution for female elderly breast cancer patients 6. However, another study conducted by Cosar et al found that elderly women with early breast cancer could significantly benefit from PRT 16. The results of a trial conducted by Vaidya et al also supported targeted radiotherapy for early breast cancer 17. The attitude towards the effect of PRT in breast cancer patients with tumor size ≤5 cm and 1-3 positive lymph nodes is still contradictory. The evidence for PRT in elderly patients is relatively weak, mainly because many clinical trials exclude women aged ≥70 years-old 2. Therefore, we conducted the study based on the SEER database to explore the role of PRT in early breast cancer.

The treatment of breast cancer depends not only on clinical pathology, but also on the breast cancer molecular subtype. Previous study revealed that molecular subtypes were also associated with radiotherapy treatment 18. Our results showed PRT was beneficial to breast cancer patients, especially in HR+ patients. However, we found no association between PRT and HR- patients. Compared with the number of HR+ patients (5754 patients), the number of HR- patients (1024 patients) was relatively insufficient. Therefore, research on larger prospective clinical trials of HR- patients was still needed to verify the conclusion.

There were also some limitations to our study. First, some potential sources of heterogeneity were inevitable. For instance, we didn't know whether those patients, who didn't receive PRT, gave up all treatment and it might lead to an exaggeration of adverse prognosis without postoperative radiotherapy. Second, we found that PRT can improve patient OS but lacks data regarding the side effects of radiotherapy in patients. Third, due to the lack of data on HER2, we couldn't analyze the role of PRT in different molecular subtypes. Fourth, most patients were white in SEER database, subgroup analysis by race may lead to improper conclusions for other race. Finally, the follow-up time is best calculated after surgery and the survival time in the SEER database is calculated from the date of diagnosis.

## Conclusions

Currently, there is still some controversy on whether early breast cancer patients with a tumor size ≤5 cm and 1-3 positive lymph nodes should undergo PRT. Although there are some limitations to our study, it is still strongly convincing with the advantages of large amounts of data. Our results showed that PRT was beneficial to elderly female patients with early breast cancer for OS, especially in HR+ patients or white patients. These findings may inform future optimized options whether elderly female patients with early breast cancer should undergo postoperative radiotherapy. Research on large prospective clinical trials is still needed.

## Supplementary Material

Supplementary tables.

## Figures and Tables

**Table 1:** Baseline Demographic and Tumor Characteristics of Unmatched Patients Stratified by Radiation Status

Characteristic	Total (6777 )	Receipt of PRT		P Value
		No (4296; 63.39)	Yes (2481; 36.61)	
**Age**				<0.001*
75-79 years	3488	2025 (47.43)	1463 (58.97)	
80-84 years	2186	1403 (32.86)	783 (31.56)	
85+ years	1103	868 (20.33)	235 (9.47)	
**Race**				0.505
Black	458	300 (7.03)	158 (6.37)	
Other	261	170 (3.98)	91 (3.67)	
White	6058	3826 (89.62)	2232 (89.96)	
**Marital Status**				<0.001*
Married	2427	1431 (33.52)	996 (40.15)	
Single (never married)	3883	2553 (59.80)	1330 (53.61)	
Widowed/Divorced/Separated	467	312 (7.31)	155 (6.25)	
**AJCC T**				<0.001*
T1	3496	1955 (45.80)	1541 (62.11)	
T2	3281	2341 (54.84)	940 (37.89)	
**Lymph node**				<0.001*
1	4199	2587 (60.60)	1612 (64.97)	
2	1679	1110 (26.00)	569 (22.93)	
3	899	599 (14.03)	300 (12.09)	
**ER**				<0.001*
Negative	1080	754 (17.66)	326 (13.14)	
Positive	4797	3542 (82.97)	1255 (50.58)	
**PR**				<0.001*
Negative	2095	1406 (32.94)	689 (27.77)	
Positive	4682	2890 (67.70)	1792 (72.23)	
**Grade**				<0.001*
Ⅰ+II	4428	2738 (64.14)	1690 (68.12)	
Ⅲ+IV	2349	1558 (36.50)	791 (31.88)	
**Laterality**				0.746
Left	3513	2220 (52.00)	1293 (52.12)	
Right	3264	2076 (48.63)	1188 (47.88)	
**Surgery**				<0.001*
Lumpectomy	2705	674 (15.7)	2031 (81.9)	
Mastectomy	4072	3622 (84.3)	450 (18.1)	

*P≤0.05 indicates statistical significance.

**Table 2:** Analysis of Overall Survival in Matched Patients Stratified by Demographic Data and Radiation Treatment

Characteristic	Patients,n	Events,n	Rate,%	Univariate Analysis		Multivariable Analysis	
				HR (95% CI)	*P* Value	HR (95% CI)	*P^a^* Value
**Age**							
75-79 years	1378	1012	73.44	Ref	Ref	Ref	Ref
80-84 years	707	593	83.88	1.39 (1.26-1.54)	<0.001*	1.37 (1.24-1.52)	<0.001*
85+ years	276	257	93.12	2.20 (1.91-2.52)	<0.001*	2.19 (1.90-2.52)	<0.001*
**Race**							
Black	154	126	81.82	Ref	Ref	Ref	Ref
Other	127	91	71.65	0.75 (0.57-0.99)	0.039	0.79 (0.60-1.04)	0.092
White	2080	1654	79.52	0.87 (0.72-1.04)	0.126	0.87 (0.73-1.05)	0.15
**Marital Status**							
Married	873	645	73.88	Ref	Ref	Ref	Ref
Widowed/Divorced/Separated	1286	1055	82.04	1.40 (1.27-1.55)	<0.001*	1.27 (1.15-1.41)	<0.001*
Single (never married)	202	162	80.20	1.28 (1.08-1.52)	0.005*	1.21 (1.02-1.44)	0.033*
**AJCC T**							
T1	1074	802	74.67	Ref	Ref	Ref	Ref
T2	1287	1060	82.36	1.37 (1.25-1.51)	<0.001*	1.25 (1.14-1.38)	<0.001*
**Lymph node**							
1	1263	963	76.25	Ref	Ref	Ref	Ref
2	632	508	80.38	1.17 (1.05-1.30)	0.005*	1.10 (0.99-1.23)	0.078
3	466	391	83.91	0.36 (1.21-1.53)	<0.001*	1.19 (1.05-1.35)	0.006*
**ER**							
Negative	378	313	82.80	Ref	Ref	Ref	Ref
Positive	1983	1549	78.11	0.76 (0.67-0.86)	<0.001*	0.96 (0.83-1.12)	0.622
**PR**							
Negative	809	671	82.94	Ref	Ref	Ref	Ref
Positive	1552	1191	76.74	0.78 (0.71-0.86)	<0.001*	0.83 (0.74-0.93)	0.001*
**Grade**							
Ⅰ+II	1494	1150	76.97	Ref	Ref	Ref	Ref
Ⅲ+IV	867	712	82.12	1.29 (1.18-1.42)	<0.001*	1.16 (1.05-1.29)	0.003*
**Laterality**							
Left	1188	942	79.29	Ref	Ref		
Right	1173	920	78.43	0.96 (0.88-1.05)	0.357		
**Surgery**							
Lumpectomy	1011	768	75.96	Ref	Ref	Ref	Ref
Mastectomy	1350	1094	81.04	1.16 (1.06-1.27)	0.001*	1.13 (1.02-1.25)	0.016*
**Radiotherapy**							
No	1574	1264	80.30	Ref	Ref	Ref	Ref
Yes	787	598	75.98	0.88 (0.79-0.96)	0.007*	0.88 (0.80-0.97)	0.01*

*P≤0.05 indicates statistical significance. *P*^a^: Adjusted for age, race, marital status, AJCC T, ER, PR, grade, surgery and radiotherapy for the multivariable COX analysis.

**Table 3:** Comparative Effectiveness of Radiation Treatment on 10- year Overall Survival by Univariate, Multivariate, and Propensity Scoree-Matched Analyses

Models	Radiotherapy	Patients	Survival Rate^a^		Univariate analysis		Multivariate analysis^b^		Propensity ScoreMatched (n ,4962)	
			5-Year (%)	10-Year (%)	Log rank χ2 test	*P* value	HR (95%CI)	*P* value	HR (95%CI)	*P* value
**All patients**	No	4296	59.71	30.56			Ref	Ref	Ref	Ref
	Yes	2491	74.3	41.5	184	<0.001*	0.84 (0.78-0.91)	<0.001*	0.88 (0.80-0.97)	0.01*
**Hormone receptor positive^c^**	No	3583	62.27	31.72						
	Yes	2171	76.6	44.9	174	<0.001*	0.80 (0.74-0.87)	<0.001*	0.84 (0.76-0.94)	0.001*
**Hormone receptor negative^d^**	No	713	46.84	24.73						
	Yes	310	50.89	30.97	7.1	0.008*	1.05 (0.87-1.27)	0.62		

^a^Unadjusted. ^b^Cox proportional hazards regression model. ^c^Estrogen receptor (ER) + and/or progesterone receptor (PR)+. ^d^ER- and PR-.*P≤0.05 indicates statistical significance.

**Table 4:** Multivariable Analysis of Overall Survival in Matched Patients Stratified by Race.

Race	PRT	Patients	Survival Rate		Univariate analysis		Multivariate analysis ^a^	
			5-year	10-year	Log rank χ2 test	*P* value	HR (95%CI)	*P* value
**White**	No	1395.00	62.55	32.87	9.21	0.002	Ref	Ref
	Yes	685.00	67.88	36.99			0.86 (0.77-0.95)	0.004
**Black**	No	96.00	54.20	26.00	1.45	0.20		
	Yes	58.00	62.00	33.60				
**Other**	No	83.00	69.90	43.30	3.51	0.06		
	Yes	44.00	53.50	27.50				

*P≤0.05 indicates statistical significance. ^a^: Adjusted for age, marital status, AJCC T, ER, PR, grade, surgery and radiotherapy for the multivariable COX analysis.

## Abbreviations

PRT: postoperative radiotherapy
SEER: Surveillance, Epidemiology, and End Results
OS: Overall Survival
NCCN: National Comprehensive Cancer Network
ER: Estrogen Receptor
PR: Progesterone Receptor
PSM: propensity score matching
HR+: hormone receptor positive
HR-: hormone receptor negative
