# Supplementary material for: Effects of Postoperative Radiotherapy in Early Breast Cancer Patients Older than 75 Years: A Propensity-Matched Analysis
Source: J Cancer. 2019 Oct 17;10(25):6225–32. doi: 10.7150/jca.35204 (PMC6856758; doi:10.7150/jca.35204)
Supplement: Supplementary file 1 — Supplementary tables. [file jcav10p6225s1.pdf]

Effects of Postoperative Radiotherapy in Early Breast Cancer Patients Older than 75 Years: A Propensity-matched Analysis

Linghui Zhou[1,2,†], Pengtao Yang[2,†], Yi Zheng[1], Tian Tian[1,2], Cong Dai[1], Meng Wang[2], Shuai Lin[2], Yujiao Deng[1,2], Qian Hao[1,2], Zhen Zhai[1,2], Hongtao Li[3], and Zhijun Dai[1]

1. Department of Breast Surgery, The First Affiliated Hospital, School of Medicine, Zhejiang University, Hangzhou 310003, China;

2. Department of Oncology, The 2nd Affiliated Hospital of Xi'an Jiaotong University, Xi'an 710004, Shaanxi, China;

3. Department of Breast Head and Neck surgery, The 3rd Affiliated Teaching Hospital of Xinjiang Medical University (Affiliated Tumor Hospital), Urumqi, 830000, China.

Correspondence: Zhijun Dai, Department of Breast Surgery, First Affiliated Hospital, Zhejiang University School of Medicine, Hangzhou 310003, China (E-Mail: dzj0911@126.com), or Hongtao Li, Department of Breast Head and Neck surgery, The 3rd Affiliated Teaching Hospital of Xinjiang Medical University (Affiliated Tumor Hospital), Urumqi, 830000, China(E-Mail: lht4656@163.com).

LH Z, PT Y and Y Z contributed equally to this work.

1. Supplemental Table S1. Baseline Demographic and Tumor Characteristics in Matched Patients Stratified by Radiation Status.

2. Supplemental Table S2. Multivariable COX Analysis of Unmatched Patients for overall survival.

Supplemental Table S1. Baseline Demographic and Tumor Characteristics in Matched Patients Stratified by Radiation Status

Characteristic	Total (2361)	Receipt of PRT		P Value
		No (1574 ; 66.6)	Yes (787; 33.3)	
Age				0.395
75-79 years	1378	924 (58.7)	454 (57.7)	
80-84 years	707	459 (29.2)	248 (31.5)	
85+ years	276	191 (9.71)	85 (10.8)	
Race				0.461
Black	154	96 (6.1)	58 (7.4)	
Other	127	83 (5.3)	44 (5.6)	
White	2080	1395 (88.6)	685 (87.0)	
Marital Status				0.917
Married	873	583 (37.0)	290 (36.8)	
Single (never married)	1286	859 (54.6)	427 (54.3)	
Widowed/Divorced/Separated	202	132 (8.4)	70 (8.9)	
AJCC T				0.458
T1	1074	698 (44.3)	376 (47.8）	
T2	1287	876 (55.7)	411 (47.8)	
Lymph node				0.486
1	5263	844 (53.6)	419 (53.2)	
2	632	411 (26.1)	221 (28.1)	
3	466	319 (20.3)	147 (18.7)	
ER				0.372
Negative	378	260 (16.5)	118(15.0)	
Positive	1983	1314 (83.5)	669 (85.0)	
PR				1.00
Negative	1011	674 (42.8)	337 (42.8)	
Positive	1350	900 (57.2)	450 (57.2)	
Grade				0.821
I+II	1494	999 (63.5)	495 (62.9)	
III+IV	867	575 (36.5)	292 (37.1)	
Laterality				0.458
Left	1188	801 (50.9)	387 (49.2)	
Right	1173	773 (49.1)	400 (50.8)	
Surgery				1.00
Lumpectomy	1011	674 (42.8)	337 (42.8)	
Mastectomy	1350	900 (57.2)	450 (57.2)	

Supplemental Table S2. Multivariable COX Analysis of Unmatched Patients for overall survival

Characteristic	Patients,n	Events,n	Rate,%	Univariate Analysis		Multivariable Analysis	
				HR (95% CI)	P Value	HR (95% CI)	P Value
Age							
75-79 years	3488	2460	70.53	Ref	Ref	Ref	Ref
80-84 years	2186	1813	82.94	1.53 (1.44-1.62)	<0.001*	1.48 (1.39-1.57)	<0.001*
85+ years	1103	1043	94.56	2.56 (2.38-2.75)	<0.001*	2.30 (2.13-2.48)	<0.001*
Race							
Black	458	360	78.60	Ref	Ref	Ref	Ref
Other	261	186	71.26	0.80 (0.67-0.95)	0.012*	0.87 (0.73-1.04)	0.125
White	6058	4770	78.74	0.95 (0.85-1.06)	0.33	1.02 (0.92-1.14)	0.682
Marital Status							
Married	2427	1789	73.71	Ref	Ref	Ref	Ref
Widowed/Divorced/Separated	3883	3146	81.02	1.34 (1.26-1.42)	<0.001*	1.14 (1.08-1.21)	<0.001*
Single (never married)	467	381	81.58	1.35 (1.21-1.51)	<0.001*	1.20 (1.07--1.34)	<0.001*
AJCC T							
T1	3496	2579	73.77	Ref	Ref	Ref	Ref
T2	3281	2737	83.42	1.47 (1.40-1.55)	<0.001*	1.26 (1.19-1.33)	<0.001*
Lymph node							
1	4199	3194	76.07	Ref	Ref	Ref	Ref
2	1679	1369	81.54	1.20 (1.12-1.27)	<0.001*	1.14 (1.07-1.21)	<0.001*
3	899	753	83.76	1.32 (1.22-1.43)	<0.001*	1.24 (1.14-1.34)	<0.001*
ER							
Negative	1080	896	82.96	Ref	Ref	Ref	Ref
Positive	4797	4420	92.14	0.74 (0.69-0.80)	<0.001*	0.93 (0.85-1.02)	0.117
PR							
Negative	2095	1719	82.05	Ref	Ref	Ref	Ref
Positive	4682	3597	76.83	0.77 (0.73-0.82)	<0.001*	0.87 (0.81-0.93)	<0.001*
Grade							
I+II	4428	3399	76.76	Ref	Ref	Ref	Ref
III+IV	2349	1917	81.61	1.27 (120-1.35)	<0.001*	1.16 (1.09-1.23)	<0.001*
Laterality							
Left	3513	2773	78.94	Ref	Ref		
Right	3264	2543	77.91	0.97 (0.92-1.02)	0.243		
Surgery							
Lumpectomy	2705	1943	71.83	Ref	Ref	Ref	Ref
Mastectomy	4072	3373	83.83	0.75 (0.70-0.81)	<0.001*	1.16 (1.07-1.25)	<0.001*
Radiotherapy							
No	4296	3543	82.47	Ref	Ref	Ref	Ref
Yes	2481	1773	71.46	0.68 (0.64-0.72)	<0.001*	0.84 (0.78-0.91)	<0.001*

*P≤0.05 indicates statistical significance. P^a: Adjusted for age, race, marital status, AJCC T, ER, PR, grade, surgery and radiotherapy for the multivariable COX analysi
